# Adherence to Therapy Using Neurostimulation Devices in the Treatment of Pediatric Attention-Deficit/Hyperactivity Disorder: Extraclinical Study

**DOI:** 10.2196/68736

**Published:** 2025-07-16

**Authors:** Allyson Calandro, Saurabh Biswas, Anthony Guiseppi-Elie

**Affiliations:** 1 Center for Bioelectronics, Biosensors and Biochips, Department of Biomedical Engineering, Texas A&M University, 400 Bizzell Street, College Station, TX, 77843, United States, 1 979-845-5532; 2 Department of Biomedical Engineering, Texas A&M University, College Station, TX, United States; 3 Department of Cardiovascular Sciences, Houston Methodist Institute for Academic Medicine, Houston, TX, United States; 4 Department of Electrical and Computer Engineering, Texas A&M University, College Station, TX, United States; 5 ABTECH Scientific, Inc., Biotechnology Research Park, Richmond, VA, United States

**Keywords:** pediatric, adolescent, ADHD, neurostimulation, therapy, adherence, real-world, extraclinical, attention-deficit/hyperactivity disorder

## Abstract

**Background:**

Pediatric and adolescent patients with attention-deficit/hyperactivity disorder (ADHD) present unique challenges in adherence to device-based therapies outside the clinical environment. The development, approval, and availability of neurostimulation devices for the treatment of ADHD have prompted extraclinical research (ie, outside the sphere of the clinic) on the real-world implementation of such therapies in a population that has difficulty remembering tasks and staying attentive to therapy.

**Objective:**

This study aims to explore the extraclinical pediatric ADHD treatment environment to ensure that design considerations and stakeholder contributions to future innovations are effective.

**Methods:**

Using the Lean LaunchPad methodology with its emphasis on customer discovery and the business model canvas, qualitative analysis methods were applied to elicit the most pertinent themes regarding ADHD treatment in children and the general perception of a new device-based treatment regimen.

**Results:**

Stakeholders expressed a desire that, for innovative ADHD therapies to appeal to children, they include a remote adherence monitoring component and maintain strong evidence of efficacy.

**Conclusions:**

Such barriers to access and desired design features should be strongly considered in the development of neurostimulation therapies for pediatric patients with ADHD. Pediatric and adolescent patients with ADHD require attentive device design considerations to achieve therapeutic adherence in a real-world setting.

## Introduction

Attention-deficit/hyperactivity disorder (ADHD) is a neuropsychiatric disorder that manifests in a pattern of inattention and/or hyperactivity-impulsivity that interferes with daily life functions [1-3]. ADHD is classified into three presentations: predominantly inattentive, predominantly hyperactive-impulsive, and a combined type, which is most common [4]. Predominantly inattentive ADHD (formerly known as attention-deficit disorder) is marked by difficulty maintaining focus or paying attention to detail [1]. Predominantly hyperactive-impulsive ADHD (traditionally known as ADHD) is marked by restlessness, fidgeting, and interrupting others [1]. Combined-type ADHD is marked by a combination of the above symptoms that varies by patient but generally involves difficulty with impulsiveness, hyperactivity, and inattention [1]. Identification and understanding of the behavior patterns of children with ADHD are vital to the pursuit of possible treatment options and accommodations [5,6].

The standard of care for pediatric ADHD varies with age and intensity of symptoms but is likely to include a combination of school accommodations, behavioral therapy, and medication [2,7,8]. For very young patients and/or patients with less-inhibiting symptoms, behavioral therapy is likely to be considered first [9]. Cognitive-behavioral therapy and behavioral parent training programs are proven therapeutic interventions shown to have positive effects in the treatment of ADHD and oppositional defiant disorder (ODD), among other comorbidities [10-13]. In the United States, school accommodations vary by child, school, and schoolteacher, but children with ADHD who meet the necessary criteria are entitled to accommodations through the federally enacted Individuals with Disabilities Education Act (IDEA) and Section 504 of the Rehabilitation Act (Section 504) [14,15]. Such school accommodations include the implementation of management strategies, such as behavioral classroom management and organizational training, and student-specific accommodations, such as extra time on tests, allowing breaks for physical movement, and detailed instructions for assignments [16]. A behavioral classroom management strategy is a well-proven and efficacious intervention that is led by the teacher to encourage positive behaviors and discourage negative behaviors [17,18]. An organizational training intervention strategy teaches the student time management, planning, and organization skills to encourage learning and reduce distractions during schoolwork [17,19]. Despite the well-studied benefits that these interventions can provide to children with ADHD, there is significant difficulty in the real-world implementation of such interventions, and the accessibility of school services varies widely across various sociodemographic groups [20-22]. Stimulant medications are the primary form of pharmacotherapy for children with ADHD, despite the considerable side effects experienced by most patients [9,23]. The most researched stimulant medications are the dopamine and norepinephrine transporter blocker, methylphenidate, which serves as the first-line pharmaceutical intervention, and amphetamines, central nervous system stimulants that serve to focus attention and improve executive function through increased release of norepinephrine and dopamine in the prefrontal cortex [24]. Methylphenidate, while a first-line pharmacotherapy for clinical ADHD, is subject to abysmal adherence [25], which has prompted growing interest in single-dose therapy [26]. Most stimulant medications exhibit a pharmacodynamic profile of a quick onset of therapeutic effects and short duration of action (varying from approximately 2‐12 h) and therefore must be taken any time focused attention and/or improved executive functioning is desired [27,28]. While school accommodations, behavioral therapy, and medication are the most studied interventions for pediatric patients with ADHD, there are still significant shortcomings in these interventions regarding accessibility, cost, and efficacy. These shortcomings can be addressed by the development of technology-based solutions, such as neurostimulation [29,30].

Recent developments in the neurological and psychological etiology of ADHD have led to increased innovations, resulting in new therapeutic interventions [2,31]. Neurostimulation is one such innovation that has begun to be developed as an alternative or adjunctive treatment for ADHD in children [31-33]. Neurostimulation, or the purposeful modulation of the nervous system’s activity, seeks to modulate brain activity and improve attention, impulse control, and executive function in children with ADHD [32]. These devices use different strategies, being invasive (eg, deep brain stimulating microelectrodes) or noninvasive (eg, transcranial stimulation including repetitive transcranial magnetic stimulation [TMS], transcranial direct current stimulation [tDCS], or external trigeminal nerve stimulation [TNS] methods. TMS noninvasively uses magnetic fields to induce electrical currents in specific regions of the brain). Typically, TMS targets the dorsolateral prefrontal cortex (DLPFC) or other relevant networks associated with ADHD symptoms. TMS has been explored for both short-term symptom management and long-term modulation of brain networks. tDCS uses low-intensity electrical currents to modulate neuronal excitability. Typically, tDCS targets the DLPFC, which is implicated in attention and impulse control. Studies show potential improvements in attention and executive function. TNS delivers mild electrical stimulation to the trigeminal nerve via electrodes placed on the forehead. TNS, Food and Drug Administration (FDA)–approved for pediatric ADHD treatment, is believed to influence arousal- and attention-regulating brain structures, such as the locus coeruleus and prefrontal cortex.

For the treatment of ADHD, tDCS and external TNS represent, even against the backdrop of small clinical studies, promising interventions [34]. tDCS has been shown to reduce clinical manifestations of ADHD and may be able to improve memory and attention performance, but remains in pilot studies and has not been approved by the FDA [33]. External TNS can be achieved noninvasively, with external electrodes and an on-body pulse generator, or invasively, with subcutaneously implanted electrodes and an implantable pulse generator [33]. External TNS transmits small electrical currents transcutaneously via supraorbital electrodes adhesively attached to the skin over the supratrochlear and supraorbital branches of the ophthalmic nerve [2]. The supraorbital branch has many connections to the brain and, when stimulated, may influence the bioavailability of electroceuticals, such as catecholamines, that potentiate ADHD symptoms [35,36]. NeuroSigma was the first company to receive FDA clearance for a neurostimulation device with a pediatric ADHD indication, called the Monarch eTNS System [37]. The Monarch device consists of a main component that generates pulses to stimulate the trigeminal nerve and an electrode array accessory to deliver the pulses [38]. It operates using radio frequency energy for over 8 hours, but the duration of treatment for each patient is determined by the physician. At present, there is no clinical evidence to support a specific timeline, frequency, or length of treatment when using Monarch. The device can deliver between 0.2 and 10.0 mA at a frequency of 120 Hz. The Monarch is battery-operated, rechargeable, and involves minimal steps to assemble and use. The kit is sold for around US $1000, with enough disposable electrode pads for 4 weeks, and additional electrode pads are sold for US $70. Neurostimulation offers pediatric patients with ADHD a promising, powerful treatment option, which is sure to gain traction as the technology develops further.

tDCS of the left and right DLPFC, using an anodal protocol, is most often used [39,40]. Safety and patent-specific or personalized stimulation parameters have not been systematically examined [41]. Using currents of 1‐2 mA applied directly to the scalp via contacting electrodes, tDCS appears effective in addressing clinical symptoms and neuropsychological deficits of patients with ADHD with no observable serious adverse effects [41]. There remains, however, considerable opportunity to address device engineering parameters such as field strength, polarity, and/or duty cycle and duration in relation to larger, appropriately diverse clinical trial cohort sizes.

The emergence of noninvasive neurostimulation technologies for the treatment of ADHD [30,33,42] heralds a new era in therapeutic options for large populations of pediatric patients [32,43]. However, stakeholders in the extraclinical (outside the sphere of the clinic) use of neurostimulators among children—parents, teachers, school nurses, and psychologists—are rarely consulted and have little opportunity for preclinical or extraclinical input into design considerations that support adherence to therapy using such devices [8,31]. As new devices and neurostimulation options are made available in the ADHD care environment, careful attention must be paid to stakeholders’ preferences, desires, and obstacles to treatment. This paper evaluates stakeholder input into design considerations in the development of neurostimulation technologies for the treatment of ADHD. While based on a small number of participants, the findings nonetheless suggest the need for further proactive engagement with the broader stakeholder community in guiding the development and clinical application of noninvasive neurostimulation for the treatment of ADHD.

## Methods

### Overview

The Lean LaunchPad is an evidence-based, experiential program created by Blank [44] that guides the testing and validation of product-focused ideas using real customer feedback. Combining principles from customer development and business model generation, the Lean LaunchPad reduces the risk of building products no one wants by focusing on validation through real-world customer interactions, pivots, and evidence-based decisions rather than assumptions. It is widely used in startup accelerators, universities, and corporate innovation programs as a practical framework for launching new product-based ventures. Customer discovery allows biodevice development teams to engage directly with potential customers to validate or invalidate their clinical use assumptions and to understand the needs, problems, and potential solutions from the customers’ perspective. The business model canvas maps key components of the business model, including value propositions, customer segments, channels, revenue streams, and more. This canvas serves as a dynamic tool that evolves based on feedback and findings from customer discovery interactions. The Lean LaunchPad is widely used by biomedical engineers in academic settings, accelerators, and incubators to help developers gain a deep understanding of their market, build products that meet real customer needs, and increase their chances of clinical success.

### Identification of Stakeholders

Identified stakeholders in the pediatric ADHD treatment environment included parents of children with ADHD, patients with ADHD, schoolteachers, school nurses, pediatric health care providers, and psychiatric health care providers. These stakeholders form a complex web of interested parties influencing adherence of children with ADHD to their prescribed treatment regimen. Interviewees were selected by drawing a 50-mile radius from the College of Medicine at Texas A&M University (Bryan–College Station area), which was subsequently extended to the whole state of Texas. Clinics and clinicians were identified from the membership of the Texas Psychological Association and the subset identified with practices that served children with ADHD. Schoolteachers, school nurses, parents, and patients were identified within the Central Texas community by the authors. At the end of each interview, interviewees were asked for referrals to another stakeholder who might offer a unique perspective, if the interviewer felt it appropriate. This served to establish a network of stakeholders with diverse backgrounds and opinions. The final stakeholder community consisted of 1 parent of 4 children with ADHD, 6 patients with ADHD, 2 schoolteachers, 4 school nurses, 8 pediatric health care providers, 4 psychiatric health care providers, and 5 additional general health care providers, for a total of 30 individuals.

### Interview Methodology

A series of questions guided by the Lean LaunchPad methodology was formulated for the semistructured interviews, with three groups of questions exploring the problem, solutions, pricing, and possible “go-to-market” strategies (Multimedia Appendix 1). The same questionnaire was used for all interviewed stakeholders to enable more rigorous analysis. Thirty stakeholders were contacted and scheduled for a ~15-minute interview via video or phone, in compliance with Texas A&M University, local, and national COVID-19 social distancing guidelines. The list of questions was sent to interviewees in advance via email. Most interviews were recorded for transcription, with interviewee consent. If consent was not granted by the interviewee for recording (2 of 30 interviewees), the interviewer took detailed notes of the conversation and question responses. This work includes answers from thirty total individuals: 4 psychiatric health care providers, 8 pediatric health care providers, 5 other health care providers, 4 school nurses, 2 schoolteachers, 6 patients, and 1 parent of 4 patients, as shown in Table 1.

Responses were transcribed and recorded in a Microsoft Excel spreadsheet by the interviewer after conclusion of the interview.

**Table 1.** Composition of the interviewed stakeholder group.

Stakeholder	Value, n (%)
Patients	6 (20.0)
Parent	1 (3.3)
Psychiatric HCP^a^	4 (13.3)
Pediatric HCP	8 (26.7)
School nurse	4 (13.3)
Schoolteacher	2 (6.7)
Other HCP	5 (16.7)

a HCP: health care professional.

### Data Analysis

Transcribed answers were subject to thematic analysis for each question. Thematic analysis is a well-established qualitative research methodology in which interview transcripts are carefully read to extract key ideas and meanings, providing a deeper understanding of the phenomenon under study. This process allowed us to identify, analyze, and interpret recurring patterns or “themes” within the dataset. The thematic analysis was conducted in three main stages: data processing, theme development, and final analysis. Data processing included transcription and initial coding. Stakeholder interviews were recorded and transcribed by the interviewer, with the responses roughly sorted by question set and question number. The first pass of analysis involved highlighting sections of text, whether words or phrases, and using a few words to describe the highlighted content, referred to here as “codes.” The full transcript of every interview was reviewed, and codes were created for any interesting, relevant, or unique information identified in the transcripts. These coded text sections were then rearranged and sorted, such that all text supporting a specific code could be viewed together. The codes created during the initial data processing stage are listed in Textbox 1.

Upon review, irrelevant and/or infrequent codes were removed from the list, and others were combined or separated as needed. Similar codes were loosely grouped together into themes, which were preliminarily named. Transcripts were then reanalyzed according to the themes, verifying that the themes did not over- or under-represent certain ideas in the data. Codes and themes were edited and adjusted as needed. The finalized themes are listed in Textbox 2.

A short definition for each theme was written to further describe how the theme was manifested in the transcripts and how it related to the larger analysis of the stakeholder investigation process.

Textbox 1. **Initial codes created during data processing.**

**Problem**

- Controlled substance
- Misuse concerns
- Cost
- Social stigma
- Forgetfulness
- Parental involvement
- Comorbidities
- Difficulty swallowing pills
- Difficulty finding a suitable medication
- Side effects from medication
- Contraindications to medication
- Multi–attention-deficit hyperactivity disorder–child families

**Solution**

- Appeal to children
- Health care professional supervision
- Parental supervision
- Evidence of effectiveness
- Brand-name medication
- Generic medication
- Behavioral therapy
- Neurofeedback therapy
- Hyperbaric therapy

**Pricing and go-to-market**

- Primary care clinic pays
- Specialty clinic pays
- Patient pays
- Price

Textbox 2. **Finalized themes created during analysis.**

**Problems**

Barriers to access

- Controlled substance and overall fear of misuse
- Costs
- Social stigma

Barriers to adherence

- Inherent attention-deficit/hyperactivity disorder traits
- Parental involvement
- Comorbidities
- Difficulty swallowing pills

Barriers to prescription

- “Trial and error” strategy
- Medication side effects

**Solution**

Desirable features

- Appeal to children
- Remote adherence monitoring (health care professional and parent)
- Evidence of effectiveness

Available and known treatment modalities

- Brand-name medication
- Generic medication
- Behavioral therapy
- Neurofeedback therapy
- Hyperbaric therapy

**Pricing and go-to-market**

Purchasing models

- Primary care model
- Specialty clinic model
- Patient purchase model

### Ethical Considerations

All participants in this study provided individual informed consent to be interviewed. All participants were deidentified and no compensation was provided for their participation. Ethical approval for this study was granted by the Texas A&M University Institutional Review Board under study number IRB2020-0898D (institutional review board title: Enhancing Therapeutic Device Adherence of Children with ADHD: An Efficacy Trial).

## Results

### Stakeholder Environment and Relationships

**Figure 1.**
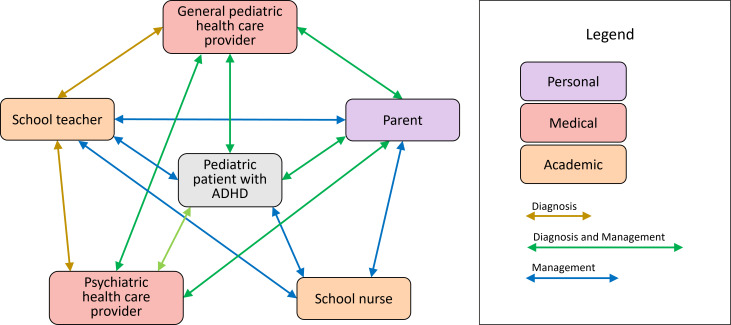
Pediatric attention-deficit/hyperactivity disorder treatment stakeholder environment, involvements, and their interactions. ADHD: attention-deficit/hyperactivity disorder.

The information gained from these interviews revealed a complex web of interactions between general pediatric health care providers, psychiatric health care providers, schoolteachers, school nurses, parents of patients, and pediatric patients, as shown in Figure 1.

### Barriers to Treatment Access

This work identified three key barriers to the access of ADHD treatment options: fear of misuse, cost, and social stigma. In addition, the work identified four key barriers to adherence to stimulant treatment: (1) inherent ADHD traits, (2) parental involvement, (3) comorbidities such as ODD [45], and (4) difficulty swallowing pills, dysphagia [46,47].

### Desired Clinical Features of Treatment Modalities

**Figure 2.**
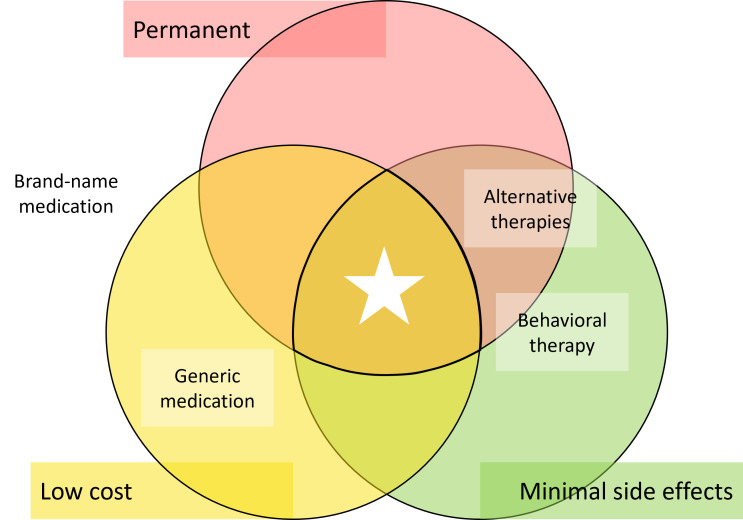
Desirable clinical features of attention-deficit/hyperactivity disorder treatment in relation to current treatment options.

Through the stakeholder investigation process, three desirable extraclinical features of a “perfect” ADHD treatment solution were identified (1) low cost, (2) permanence, and (3) minimal side effects. The Venn diagram of Figure 2 schematically illustrates the balance of these features among the various treatment options.

### Desired Usability Features of Treatment Modalities

Two desirable usability features for future treatment modalities were elucidated after a minimal introduction to neurostimulation devices, which are as follows: (1) appeal to children and (2) adherence monitoring.

### Device-Based Treatment Purchase Options

**Figure 3.**
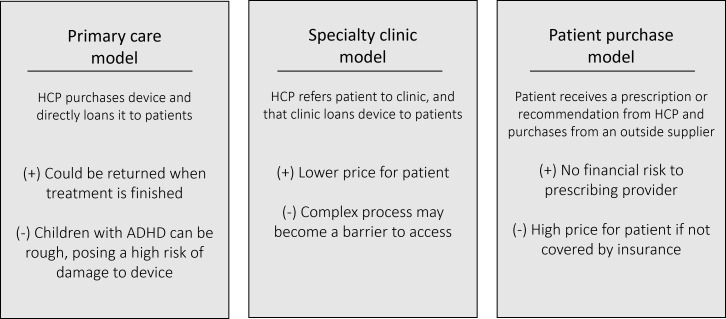
Purchase option models proposed and presented to stakeholders. ADHD: attention-deficit/hyperactivity disorder; HCP: health care professional.

Of the 3 therapeutic access models presented to stakeholders, shown in Figure 3, the patient purchase model was the most popular among the interviewed stakeholders.

### Identified Advantageous Entry Markets

Three highly specific entry markets were identified: (1) multi-ADHD-child families, (2) families desiring conservative treatment modalities, (3) patients requiring brand-name medication, and (4) patients with contraindications to stimulants.

## Discussion

### Stakeholder Environment and Relationships

Each party (Figure 1) plays a specific role in the diagnosis and management of pediatric ADHD, often interacting with one another and influencing each other’s actions. Generally speaking, a diagnosis is made upon self-reporting to either a general pediatric health care provider or a psychiatric health care provider by the patient, their parents, and/or their schoolteacher. Once a diagnosis is made, the patient is clinically managed by their parents, health care providers, and sometimes, the school nurse. Some patients are diagnosed and managed exclusively by a general pediatric health care provider, some exclusively by a psychiatric health care provider, and some are diagnosed and managed concomitantly by a general pediatric health care provider and a psychiatric health care provider. This largely depends on the comfort level of the general pediatric health care provider with pediatric psychiatry and the presence of possible comorbidities that may complicate the process and be better handled by a psychiatric professional. Health care providers involved with a specific patient are responsible for clinical management and the administration of pharmacotherapy. The schoolteacher is responsible for managing the patient’s behavior in the classroom, while the school nurse oversees behavioral management in the broader school environment outside the classroom. If the patient is on immediate-release stimulant pharmacotherapy, the schoolteacher is generally the first to notice a missed dose on a specific day, as their behavior will be affected when the child arrives at school. This is where the school nurse’s responsibilities become apparent, including administering medication for patients requiring mid-day dosing and managing side effects that occur during school hours. Finally, the parents of a pediatric patient with ADHD are largely responsible for all the activities surrounding treatment, as they advocate for and represent their child throughout the process. All these parties must be in regular communication with one another to ensure optimal diagnosis and clinical management of a pediatric patient with ADHD.

### Barriers to Treatment Access

The three key barriers to access to ADHD treatment options—fear of misuse, cost, and social stigma—have pragmatic origins. Health care providers and parents alike are often concerned about possible misuse of stimulant medication, given that most are schedule II controlled substances [48]. Because of this classification, health care providers, under normal circumstances, cannot prescribe more than 30 days’ medication at a time and must follow up with each patient approximately once per month [49]. This research perceived a significant fear of misuse among prescribing physicians and parents alike. Another identified barrier to access to stimulant medications is cost. Depending on which type of medication is prescribed and is found to work well for the patient, the cost of monthly medication can, in many cases, be prohibitive. This can be ameliorated by seeking insurance coverage for a specific stimulant medication or circumvented altogether by finding an alternative medication option that is already covered by the patient’s insurance. One general pediatric health care provider stated that “it is hard to find a prescription and medication that is effective and helpful for the patient, while also being covered by insurance.” An additional barrier to access to stimulant medications is the perceived social stigma surrounding their use. This theme was most prominent among interviews with general pediatric health care providers, as several mentioned situations in which they felt a patient was a great candidate for stimulant medication, but the parents of the patient were extraordinarily hesitant to start their child on stimulants. One physician elaborated on the familial strife this can cause, noting that when parents hesitate to start their child on stimulants, it “creates frustration and difficulties for the patients themselves.” Only one physician was able to pinpoint what exactly she felt was causing the social stigma, stating that it was “from fear that the stimulants could cause addiction problems later.” There is substantial evidence in the literature showing no such association between stimulant use and substance use disorder in patients with ADHD, even when stimulant treatment is initiated in childhood [50-55]. More research must be done and more effective communication engendered to further clarify the absence of association between stimulant medication and substance use disorder comorbidity in ADHD.

Four key barriers to adherence to stimulant treatment were identified during the stakeholder investigation process, including inherent ADHD traits, parental involvement, comorbidities such as ODD [45], and difficulty swallowing pills, dysphagia [46,47]. Patients with ADHD are, by definition, inattentive, hyperactive, and impulsive, and thus do not have favorable traits for adhering well to strict medication schedules. A patient’s adherence to stimulant medication varies from patient to patient and is found to be highly dependent on the level of parental involvement that the patient experiences. The importance of parental involvement was mentioned 16 times throughout the interviews, most stating that “compliance [to medication] without parents is extremely low…with parents, the number [adherence rate] is higher.” A noteworthy point is that, because of ADHD’s highly heritable nature, the possibility of a parent of a patient having ADHD is higher than average [56,57]. Therefore, adherence to medication can be affected not only by the child’s condition, but also by that of the engaged parent. Comorbidities to ADHD were also frequently mentioned in the interviews, including obsessive-compulsive disorder, autism spectrum disorder, ODD, and bipolar disorder. These findings are consistent with the literature on common comorbidities with ADHD [58]. Specifically, ODD can create difficulty with adherence to treatment, as patients with ODD are inherently defiant of authority and argumentative [1].

There are also barriers to finding an effective and tolerable medication type and dosage. Patients and physicians expressed that identifying the most effective medication and dosage for the patient’s specific ADHD symptoms was extremely difficult. The most common strategy was simply “trial and error” with medications, in some cases taking several years before a suitable medication and dosage were found. The therapeutic window for most stimulants is narrow, with the optimal dosage often laborious to find for each patient, and the increase in side effect severity occurring rapidly outside the therapeutic window. Stimulant side effects were the most frequently mentioned topic in this study, with 43 mentions overall. The side effects of stimulant medication can be harsh and can significantly complicate ADHD management, particularly in children. One adolescent patient stated:

*The side effects can really impact your life. I’ve been taking medication for years, since elementary [school]. I know in elementary [school], some people would ask me if I was mad at them because I was not as creative or expressive as before. The medicine sometimes doesn’t make you feel like yourself.*

This theme, of stimulant medication causing a patient to not “feel like themselves,” was echoed by many of the young adults with ADHD interviewed. They were quite sincere in their discussion about the side effects they experienced and discussed how impactful this feeling had been in their social and familial relationships.

### Desired Clinical Features of Treatment Modalities

The three desirable clinical features of a “perfect” ADHD treatment solution—low cost, permanence, and minimal side effects—are partially met by the most common existing treatment options: pharmacotherapy [7], behavioral therapy [59], and complementary and alternative therapies [60]. Each of the common treatment options addresses some of these desirable features, but none addresses all three, as shown in the Venn diagram of Figure 2. A distinction is drawn between generic and brand-name medication options due to the drastic difference in cost to the patient.

Brand-name medication is neither permanent nor low-cost and has significant side effects. Generic medication may be low-cost, but it is also not permanent and may have significant side effects. Behavioral therapies have minimal side effects and can be permanent but are generally not and can be quite costly for patients. Alternative therapies can claim to be more permanent with less side effects but are very high cost, and generally the effectiveness is not well-proven in the literature [61]. Alternative therapies included in this work are hyperbaric oxygen therapy and neurofeedback therapy. Despite its lack of the key desirable features identified above, stimulant medication is the most prominent and popular treatment option discussed in these stakeholder interviews. Neurostimulation devices could meet these desired clinical features, as the modality is developed to be permanent with minimal side effects, and over time, could be offered at a low cost over the lifetime of the device and the duration of therapy [2].

### Desired Usability Features of Treatment Modalities

Neurostimulation therapy, notably tDCS, for example, requires wearing a headset for 20 minutes each day for 10 consecutive days, which was disclosed to participants. Two desirable usability features for future treatment modalities were elucidated after this minimal introduction to neurostimulation devices: (1) appeal to children and (2) adherence monitoring. Many stakeholders discussed the need for treatment to be appealing to children in some way to engage them in the treatment process and consequently, ensure ownership of the therapy. One schoolteacher stated the following insight on the importance of appeal to children:

*It depends on how it is presented. Some kids do not even like glasses, [because] they want it to be seen as ”cool.” They [children with ADHD] would probably be more apt if they could wear it [a novel treatment device] with a hat. Kids are likely willing to try.*

Two of the psychiatric health care providers echoed this sentiment, stating the vital need for emotional, creative, or imaginative appeal for children with ADHD to engage with the device. These observations point to the need for design features of color, texture, and form factor that promote, rather than dissuade, engagement with the therapeutic device [8]. An important consideration was that electrodes contacting the scalp should be designed to make contact through hair of various types and textures. This suggests the use of brush-type electrodes with bristles of conductivity to deliver the stimulating current and sufficient elastic modulus sufficient to penetrate hair of multiple textures to contact the scalp [62-64]. Suitable electrical conductivity is typically achieved using an electrolyte-filled sponge to first bathe the electrodes. New electrode materials that permanently retain electrolyte can render the system “plug-and-play,” without the need for preparing the electrodes with saline-soaked sponges. An additional feature requested by general pediatric health care providers and patients was that of remote adherence monitoring. Physicians reported appreciating the compliance estimate made available by looking at pharmacy refill requests and expressed interest in seeing data on patient usage for a treatment device in the home. Patients also stated that they would prefer for their clinician to “closely monitor [their] treatment and usage of the device.” Both an appeal to children and an adherence monitoring system should be closely considered in the usability development of upcoming pediatric ADHD treatment modalities [8].

### Device-Based Treatment Purchase Options

The primary care model shows that a primary care provider or general practice clinic purchases the device and then supplies it to its patients for the duration of the prescription. The largest advantages to this loaner model are that the device could be returned when treatment was finished, likely reducing the overall cost of treatment for the patients, and the device could be sanitized and reissued for use. The largest drawback to this model is that children with ADHD are generally hyperactive and impulsive, thus increasing the risk of damage to the device while it is loaned out to the patient. This incurs difficulty on the part of the primary care physician, as the devices may quickly become damaged and/or unusable. The specialty clinic model shows that when a primary care provider identifies a patient as a good candidate for neurostimulation treatment, they refer that patient to an outside clinic, which manages the treatment course and loans devices out to patients. The most significant benefit of this model is that it could reduce the price of treatment to patients and remove some of the responsibility of implementing new technology from the primary care provider. The most significant disadvantage to this model is that the added step in the process may unnecessarily complicate the system and may become a barrier to access for patients. The patient purchase model shows that a patient receives a prescription for neurostimulation treatment from their health care provider and then purchases the device themselves from an outside supplier. The most compelling advantage to this model is that it requires no financial risk on the part of the prescribing provider, increasing the likelihood of the device being prescribed overall. The most compelling disadvantage to this model is the high cost, which is the full responsibility of the patient. Despite this point, the patient purchase model was the most popular among interviewed stakeholders because of the ease of accessibility to treatment and the low financial risk required from providers.

**Figure 4.**
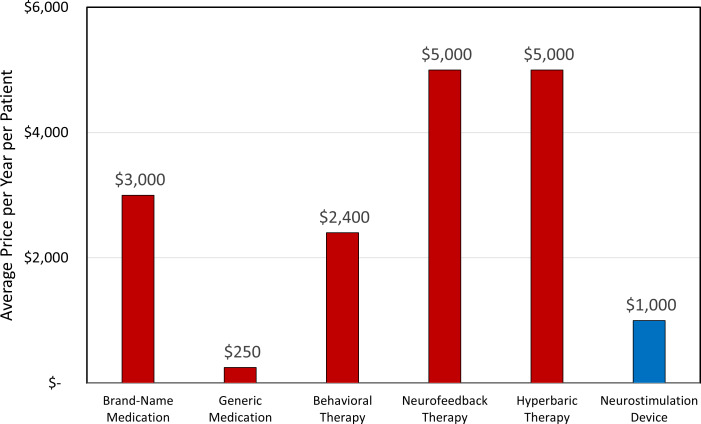
Reported average price (in US dollars) per year of selected attention-deficit/hyperactivity disorder treatment modalities and projected price (in US dollars) per intervention using neurostimulation device therapy.

Interviewees reported their perception of the average price of various ADHD treatments with which they were familiar. These estimates were analyzed and distilled into five categories: brand-name medication, generic medication, behavioral therapy, neurofeedback, and hyperbaric therapy, as shown in maroon in Figure 4. The interviewees were also asked to report approximately what price they thought was appropriate for one round of neurostimulation treatment for ADHD, which is shown in blue in Figure 4.

### Identified Advantageous Entry Markets

Throughout the analysis of the stakeholder interviews, a few key advantageous entry markets were revealed. These highly specific entry markets have unique pain points that are neglected by current pediatric ADHD treatment modalities. These markets include multi-ADHD-child families, families desiring conservative treatment modalities, patients requiring brand-name medication, and patients with contraindications to stimulants. Multi-ADHD-child families are somewhat common due to the high heritability of ADHD. Over time, neurostimulation could prove to be a highly cost-effective option for families requiring pharmacotherapy for several children over many years. Many stakeholders expressed concern about the large population of families who feel a social stigma surrounding stimulant medication and generally desire more conservative treatment for their children. A neurostimulation device could offer them effective treatment without the use of stimulant medication. Patients who require brand-name medication would also be a key entry market, as brand-name stimulant medications carry extremely high costs for patients. The final key entry market identified in this study is patients for whom stimulant medication is contraindicated, including those with symptomatic cardiovascular disease, hyperthyroidism, hypertension, and/or a history of substance use disorder, among other things [65,66]. For these patients, there are currently very few options of any kind for long-term, effective management of ADHD. The population with pharmacological contraindications is a primary entry market for neurostimulation devices for the treatment of pediatric ADHD.

### Limitations of the Study

This study draws upon a relatively small number of stakeholders, but an otherwise broad set of representative stakeholders in ADHD therapy. Extrapolating from this small data set is one limitation. Second, the stakeholders are from a region, initially within a 50-mile radius of the College of Medicine at Texas A&M University and eventually expanding to the entire State of Texas. There may be a regional predisposition toward technology interventions among practitioners who work near a research-intensive, engineering-centric university. The underwhelming participation by parents, an important stakeholder group in ADHD therapy, is noteworthy. Finally, participants were purposively selected rather than randomly sampled.

### Future Work

The stakeholder engagement results could be strengthened with the addition of more interviews, particularly those with parents and/or guardians of children with ADHD, as the children themselves are not particularly available for or willing to complete such interviews. In addition, another round of interviews could be conducted with more precise questions to elucidate more exact pain points, desired solutions, and purchase options. Both supplementary propositions would require additional time, effort, and funding that were out of the scope of this body of work.

### Conclusions

A customer discovery process, as outlined by the National Science Foundation Innovation-Corps, was designed and executed to investigate the pediatric ADHD treatment environment. Thirty stakeholders were interviewed using semistructured interview methodology, and their responses were recorded and analyzed for key themes and insights. A specific interest was placed on the developing technology of neurostimulation for the treatment of pediatric ADHD, and the stakeholders gave insightful feedback on problems in the pediatric ADHD treatment environment, important desired features to be considered in the development of a new treatment modality, and purchase option modeling for the distribution of neurostimulation devices throughout the market. These sessions revealed a complex web of stakeholders involved in the diagnostic and therapeutic management of a child with ADHD. Numerous key barriers to treatment access were identified, clarifying the difficulty that stakeholders face when choosing a treatment regimen. A major “pain-point” for pediatric patients with ADHD in their current treatment options was identified in the side effects, high cost, and impermanence, which could be mitigated with the development of therapeutic neurostimulation devices. Stakeholders desired a new treatment modality that had specific usability features of (1) a creative or social appeal to children, and (2) an adherence monitoring system. Investigation regarding the stakeholder-desired purchase method of a device-based treatment modality revealed that a patient purchase model was the most popular because of the ease of accessibility to treatment and the low financial risk required from prescribing providers. Several advantageous entry markets were identified for a device-based pediatric ADHD treatment option, including multi-ADHD-child families, families desiring conservative treatment modalities, patients requiring brand-name medication, and patients with contraindications to stimulants. Overall, this work revealed a niche need for the development of a new ADHD treatment modality, and neurostimulation appears to be a hopeful option in this regard.

## Supplementary material

10.2196/68736 Multimedia Appendix 1 Stakeholder interview questions.

## Abbreviations

ADHD: attention-deficit/hyperactivity disorder
DLPFC: dorsolateral prefrontal cortex
FDA: Food and Drug Administration
IDEA: Individuals with Disabilities Education Act
ODD: oppositional defiant disorder
tDCS: transcranial direct current stimulation
TMS: transcranial magnetic stimulation
TNS: trigeminal nerve stimulation
