# Supplementary material for: Adherence to Therapy Using Neurostimulation Devices in the Treatment of Pediatric Attention-Deficit/Hyperactivity Disorder: Extraclinical Study
Source: JMIR Neurotechnol. 2025 Jul 16;4:e68736. doi: 10.2196/68736 (PMC12671317; doi:10.2196/68736)
Supplement: Multimedia Appendix 1 [file neuro-v4-e68736-s001.docx]

Supplemental

Extraclinical Adherence Considerations for the Development of Neurostimulation Devices in the Treatment of Pediatric ADHD

Allyson Camp ^1,2^, Saurabh Biswas ^2^ and Anthony Guiseppi-Elie ^1,3,4,5,^*

^1^ Center for Bioelectronics, Biosensors and Biochips (C3B®), Department of Biomedical Engineering, Texas A&M University, 400 Bizzell St, College Station, TX 77843, United States

^2^ Department of Biomedical Engineering, Texas A&M University, 400 Bizzell St, College Station, TX 77843, United States

^3^ Department of Electrical and Computer Engineering, Texas A&M University, 400 Bizzell St, College Station, TX 77843, United States

^4^ Department of Cardiovascular Sciences, Houston Methodist Institute for Academic Medicine and Houston Methodist Research Institute, 6670 Bertner Ave., Houston, TX 77030, United States.

^5^ABTECH Scientific, Inc., Biotechnology Research Park, 800 East Leigh Street, Richmond, VA 23219, United States

*****Correspondence: guiseppi@tamu.edu; Tel.: +1(804)347.9363; Fax: +1(8044)347.9363

Academic Editor: *name*

Received: date; Accepted: date; Published: date

Allyson Camp <[allysoncamp@tamu.edu](mailto:allysoncamp@tamu.edu)> : 0000-0003-3832-5946

Saurabh Biswas <[saurabh_biswas@tamu.edu](mailto:saurabh_biswas@tamu.edu)>; 0000-0002-2413-7170

Anthony Guiseppi-Elie <[guiseppi@tamu.edu](mailto:guiseppi@tamu.edu)> : 0000-0003-3218-9285

**IRB Study number:** IRB2020-0898D

**IRB Title:** Enhancing Therapeutic Device Adherence of Children with ADHD: An Efficacy Trial

Supplemental Material

Stakeholder Interview Questions

Problem: ADHD Treatment, Adherence Questions

1. Are you satisfied with the current ADHD treatments available to you?
2. What are the difficulties related to those current treatments from the patient’s standpoint? From your standpoint?
3. With the current treatments, what do you feel the compliance rate is to your treatments?

Solution: Device Description Questions

*The FDA has provided approval for neurostimulation devices for the treatment of various psychological disorders. We are especially interested in how neurostimulation devices for ADHD could be implemented in the clinic.* *Neurostimulation therapy, notably tDCS, requires wearing a head set for 20 minutes each day for 10 consecutive days.*

1. Do you think a device for stimulation for ADHD would be well-received among your patients? Your coworkers?
2. How do you think a device like that would impact or meet your criticisms of other treatment options?
3. If you were using a medical device as a psychiatric treatment for pediatric ADHD patients, would your clinic buy the device and lease/lend it out to patients? Or would patients purchase the devices individually from your office/or supplier?

Price & Go-to-Market: Payment Option Questions

1. Do psychiatric services fall in-network?
2. Given your experience, what would be a reasonable price for this therapy?
3. Do you think leasing devices from your clinic or having the patient purchase the device would affect compliance and adherence for treatment?
4. Do you think patients would be willing to purchase the device or pay for its treatment if the insurance does not cover the cost?
5. What are the current costs for drug therapy/prescriptions for patients currently using medication as a treatment for ADHD?

Concluding Questions

1. What do you wish I had asked?
2. Is there someone else I should talk to? Would you be willing to make an introduction?
